# Critical incidents during anesthesia: prospective audit

**DOI:** 10.1186/s12871-023-02171-4

**Published:** 2023-06-14

**Authors:** K. Bielka, I. Kuchyn, M. Frank, I. Sirenko, A. Yurovich, D. Slipukha, I. Lisnyy, S. Soliaryk, G. Posternak

**Affiliations:** 1 Bogomolets National Medical University, department of surgery, anesthesiology and intensive care of postgraduate education, Kyiv, Ukraine; 2 Mukachevo Central District Hospital (St. Martin Hospital), Mukachevo, Ukraine; 3 Medical center “Medion”, Poltava, Ukraine

**Keywords:** Anesthetic emergencies, Patient safety, Incident reporting, Performance analysis, Reporting environment

## Abstract

**Background:**

Critical incident reporting and analysis is one of the key components of patient safety in anesthesiology. The aim of this study was to determine the frequency and characteristics of critical incidents during anesthesia, main causes and factors involved, influence on patient outcomes, prevalence of incident reporting and further analysis.

**Methods:**

A multicenter prospective audit was conducted at the clinical departments of the Bogomolets National Medical University during the period from 1 to 2021 to 1 December 2021. 13 hospitals from different Ukrainian regions took part in the study. Anesthesiologists voluntarily submitted critical incident reports into a Google form as they occurred during the working shifts, reporting the details of the incident, and the incident registration routine in their hospital. The study design was approved by the Bogomolets National Medical University (NMU) ethics committee, protocol #148, 07.09.2021.

**Results:**

The incidence of critical incidents was 9.35 cases per 1000 anesthetic procedures. Most common incidents were related to the respiratory system: difficult airway (26.8%), reintubation (6.4%), oxygen desaturation (13.8%); cardiovascular system: hypotension (14.9%), tachycardia (6.4%), bradycardia (11.7%), hypertension (5.3%), collapse (3.2%); massive hemorrhage (17%). Factors associated with critical incidents were elective surgery (OR 4.8 [3.1–7.5]), age from 45 to 75 years (OR 1.67 [1.1–2.5]), ASA II (OR 38 [13–106]}, III (OR 34 [12–98]) or IV (3.7 [1.2–11]) compared to ASA I; regional anesthesia (OR 0.67 95 CI 0.5–0.9) or general anesthesia (GA) and regional anesthesia combination (OR 0.55 95 CI 0.3–0.9] decreased the risk of incidents compared to GA alone. Procedural sedation was associated with increased risk of a critical incident, compared to GA (OR 0.55 95 CI 0.3–0.9). The incidents occurred most commonly during the maintenance phase (75/113, 40%, OR compared to extubation phase 20 95 CI 8–48) or the induction phases of anesthesia (70/118, 37%, OR compared to extubation phase 18 95 CI 7–43). Among common reasons that could lead to the incident, the physicians have identified: individual patient features (47%), surgical tactics (18%), anesthesia technique (16%) and human factor (12%). The most frequent failings contributing to the incident occurrence were: insufficient preoperative assessment (44%), incorrect interpretation of the patients’ state (33%), faulty manipulation technique (14%), miscommunication with a surgical team (13%) and delay in emergency care (10%). Furthermore, 48% of cases, as judged by participating physicians, were preventable and the consequences of another 18% could be minimized. The consequences of the incidents were insignificant in over a half of the cases, but in 24.5% have led to prolonged hospital stay, in 16% patients required an urgent transfer to the ICU and 3% of patients died during their hospital stay. The majority of the critical incidents (84%) were reported through the hospital reporting system, using mostly paper forms (65%), oral reports (15%) and an electronic database (4%).

**Conclusion:**

Critical incidents during anesthesia occur rather often, mainly during the induction or maintenance phases of anesthesia, and could lead to prolonged hospital stay, unplanned transfer to the ICU or death. Reporting and further analysis of the incident are crucial, so we should continue to develop the web-based reporting systems on both local and national levels.

**Study registration:**

NCT05435287, clinicaltrials.gov, 23/6/2022.

## Background

The key principle in decreasing the healthcare-associated risks is to report and analyze things that went wrong [1].

In anesthesia it could be achieved via critical incident reporting, which is one of the key components of the Helsinki Declaration on Patient Safety in Anesthesiology [2]. A critical incident in anesthesia is defined as an untoward and preventable mistake, which leads to, or could have led to a negative patient outcome [3]. Identification of risk factors, causes and circumstances of critical incident occurrence could help to prevent them. Furthermore, learning the consequences of these incidents would prove their impact on patient outcomes and recovery.

While we have several studies on critical incidents during anesthesia in high-income countries [4–6], there is very limited information available about these incidents, as well as healthcare-related harm, occurring in low- and middle-income counties [7].

The aim of this study was to determine the frequency and characteristics of critical incidents during anesthesia, main causes and factors involved, influence on patients outcomes, prevalence of emergency notification and response systems used in hospitals.

## Materials and methods

A multicenter prospective audit was conducted at the clinical departments of Bogomolets National Medical University (postgraduate department of surgery, anesthesiology and intensive care). A total of 13 hospitals took part in the prospective cohort study: Kyiv City Clinical Hospitals #1, #4, #17, University Clinic, Kyiv City Maternity Hospital #5, Shalimov National Institute of Surgery and Transplantology, National Cancer Institute, medical center “Oberig”, medical center “Medion” in Poltava, Saint Martin Hospital in Mukachevo, Vinnytsia City Clinical Emergency Hospital, Amosov National Institute of Cardiovascular Surgery, “Into-Sana” medical center in Odesa.

The data were collected during 6 months (from 1 to 2021 to 1 December 2021). The study design was approved by the ethics committee of the Bogomolets National Medical University (protocol #148, 07.09.2021). It was retrospectively registered at clinicaltrials.gov on 23/06/2022 (NCT05435287).

Primary objective of the audit was to evaluate the incidence of critical incidents during anesthesia, and to identify their possible causes and risk factors. Secondary objectives were to assess the critical incident influence on patients’ outcomes, as well as prevalence of emergency notification, and response systems used in hospitals.

At each clinical center there was an investigation coordinator, who spread the printed information about the study design with a QR-code of the study link [8]. The data collection audit design was also explained to all anesthetists at weekly department meeting. They were asked to anonymously submit any critical incidents if they occurred during the working shift into a Google form, and report the details of the incident, circumstances of its occurrence, possible risk factors or causes, medical consequences, opinion on whether it could be prevented, and what measures could help to prevent it next time. There was no need to report the patients’ and the anesthesiologists’ individual information (Name, Surname, hospital identification number). The QR-code to the form was available in all operating rooms and anesthetists were regularly reminded to report any critical incident. Blinding and randomization were not carried out.

The types of critical incidents (inclusion criteria) were:

### Airway

difficult intubation, unsuccessful intubation, reintubation, inadvertent esophageal intubation, difficult mask ventilation or laryngeal mask insertion, inadvertent extubation, bronchospasm, laryngospasm, oxygen desaturation < 90%, hypo-/hypercapnia, pneumothorax, aspiration.

### Cardiovascular

hypotension (systolic AP < 70), bradycardia (HR < 40/min), tachycardia (HR > 140/min), tachyarrhythmia, hypertension (systolic AP > 200), cardiogenic lung edema, acute myocardial ischemia, cardiac arrest, hemolytic transfusion reaction, massive hemorrhage > 1000ml, air embolism, collapse.

### Regional anesthesia

wrong drug administration, total spinal block, systemic LA toxicity, paresthesia, nerve damage, intraneural injection.

### Medication

prescription error, allergy, anaphylaxis, missed dose, side effects, malignant hypertension.

### Equipment

laryngoscope or videolaryngoscope malfunction, circuit leak, absence of the absorbent etc.

### Other

responders had an option to write any other critical incident which was not mentioned above.

We only included cases of patients between the ages of 18 and 75 years old.

### Statistical analysis

Sample size was calculated using MedCalc Software version 16.8.4 (MedCalc Software bvba, Acacialaan 22, 8400 Ostend, Belgium). Based on the incidence of critical incidents, reported by other studies, they appeared as frequently as 4,5 to 65 cases per 1000 anesthesias [9–11]. Analysis was performed using IBM SPSS Statistics for Windows, Version 26.0. Armonk, NY: IBM Corp. Numerical data are presented as medians and 25–75 quartiles. Simple descriptive statistics are provided for discrete numerical data, odds ratio were calculated for possible risk factors. The probability of error (p) was considered insignificant at p < 0.05.

## Results

A total of 188 incidents was registered in a period of six months. During that time, approximately 20,100 procedures under anesthesia were performed in the 13 participating hospitals. The incidence of critical incidents was 9.35 cases per 1000 anesthetic procedures. The median patient’s age was 62 [44–75] years. Detailed information on factors associated with critical incidents is provided in the Table 1.

Factors associated with critical incidents (Table 2) were ages between 45 and 75 years (OR 1.67 [1.1–2.5]), ASA II (OR 38 [13–106]}, III (OR 34 [12–98]) or IV (3.7 [1.2–11]) compared to ASA I; regional anesthesia (OR 0.67 95 CI 0.5–0.9) or general anesthesia (GA) and regional anesthesia combination (OR 0.55 95 CI 0.3–0.9] decreased the risk of incidents compared to GA alone]. Procedural sedation was associated with increased risk of critical incident occurrence, compared to GA (OR 0.55 95 CI 0.3–0.9).

**Fig. 1:**
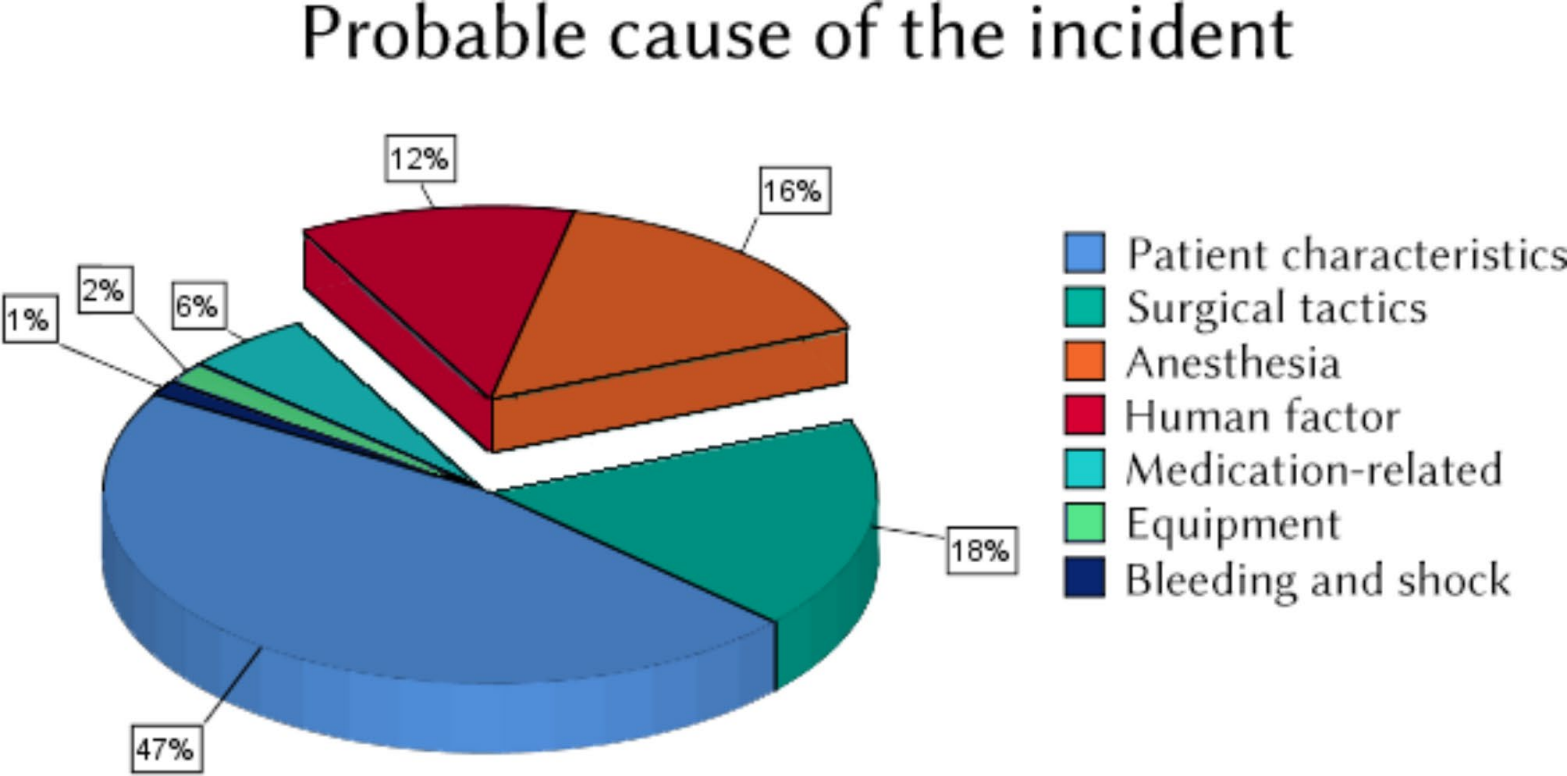
Probable cause of the critical incident, according to reporting physicians (multiple choice question)

No cases of peripheral nerve damage, paresthesia, wrong drug administration or intraneural injection were reported. Probable causes of the incident as judged by the reporting physician are described in Fig. 1.

**Table 1:** Registered critical incidents

	Type of critical incident	n (%)
AIRWAY	Difficult intubation	36 (19.1)
	Unsuccessful intubation	6 (3.2)
	Vasopressors	197
	Reintubation	12 (6.4)
	Esophageal intubation	4 (2.1)
	Difficult mask ventilation	9 (4.8)
	Unintentional extubation	2 (1.1)
	Bronchospasm	6 (3.2)
	Laryngospasm	6 (3.2)
	Oxygen desaturation (spO_2_ < 90%)	26 (13.8)
	Pneumothorax	4 (2.1)
	Gastric content aspiration	4 (2.1)
HEMODYNAMIC	Hypotension (SAP < 70mmHg)	28 (14.9)
	Hypertension (SAP > 200mmHg)	10 (5.3)
	Bradycardia (HR < 40 bpm)	22 (11.7)
	Tachycardia (HR > 140 bpm)	12 (6.4)
	Cardiogenic pulmonary edema	2 (1.1)
	Cardiac arrest	6 (3.2)
	Massive hemorrhage (> 1000ml)	32 (17)
	Collapse	7 (3.7)
	Arrhythmia	3 (1.6)
	IVC compression syndrome	2 (1.1)
OTHER	Malignant hyperthermia	1 (0.5)
	Ventilator circuit leak	3 (1.6)
	Total spinal block	4 (2.1)
	Unintentional dural puncture	2 (1.1)
	Anaphylactic shock	4 (2.1)

*spO*_*2*_ *– peripheral oxygen saturation; SAP – systolic arterial pressure; bpm – beats per minute; HR – heart rate; IVC – inferior vena cava*

**Table 2:** Factors associated with critical incidents

Factor	N (%)	OR 95 CI	p
Age 18–44	78 (41)	OR 1.67 [1.1–2.5]	p = 0.01
Age 45–75	102 (54)		
Female	102 (54)	OR 0.7 [0.1 − 0.09]	p = 0.12
Male	86 (45)		
ASA I	4 (2)	-	
ASA II	85 (45)	OR 38 [13–106]	p < 0.001
ASA III	81 (43)	OR 34 [12–98]	p < 0.001
ASA IV	14 (7)	OR 3.7 [1.2–11]	p = 0.03
ASA V	4 (2)	OR = 1	p = 1
Elective surgery	100 (53)	OR 1.5 [0.98–2.2]	p = 0.07
Urgent surgery	70 (37)		
General anesthesia	72 (38)	-	
Regional anesthesia	65 (34)	OR 0.67 [0.5–0.9]	p = 0.028
General + regional anesthesia	23 (12)	OR 0.55 [0.3–0.9]	p = 0.015
Procedural sedation	28 (14)	OR 2.4 [1.5–3.7]	p < 0.001
Induction phase of anesthesia	70 (43)	OR 6 [3.4–10.8]	p < 0.001
Maintenance phase of anethesia	75 (46)	OR 6.8 [3.8–12.2]	p < 0.001
Extubation phase of anesthesia	18 (11)	-	-

ASA - American Society of Anesthesiologists physical status score.

In 138 (73.4%) cases the critical incident occurred during the interval between 8 AM and 5 PM on weekdays. 25 (13.8%) incidents took place on holidays and weekends during the same interval and 25 (13.8%) occurred during the interval between 5 PM and 8 AM.

The incidents occurred most commonly during the maintenance phase, compared to extubation phase (OR 6.8 95% CI 3.8–12.2), or the induction phases of anesthesia compared to extubation phase (OR 6; 95% CI 3.4–10.8).

**Fig. 2:**
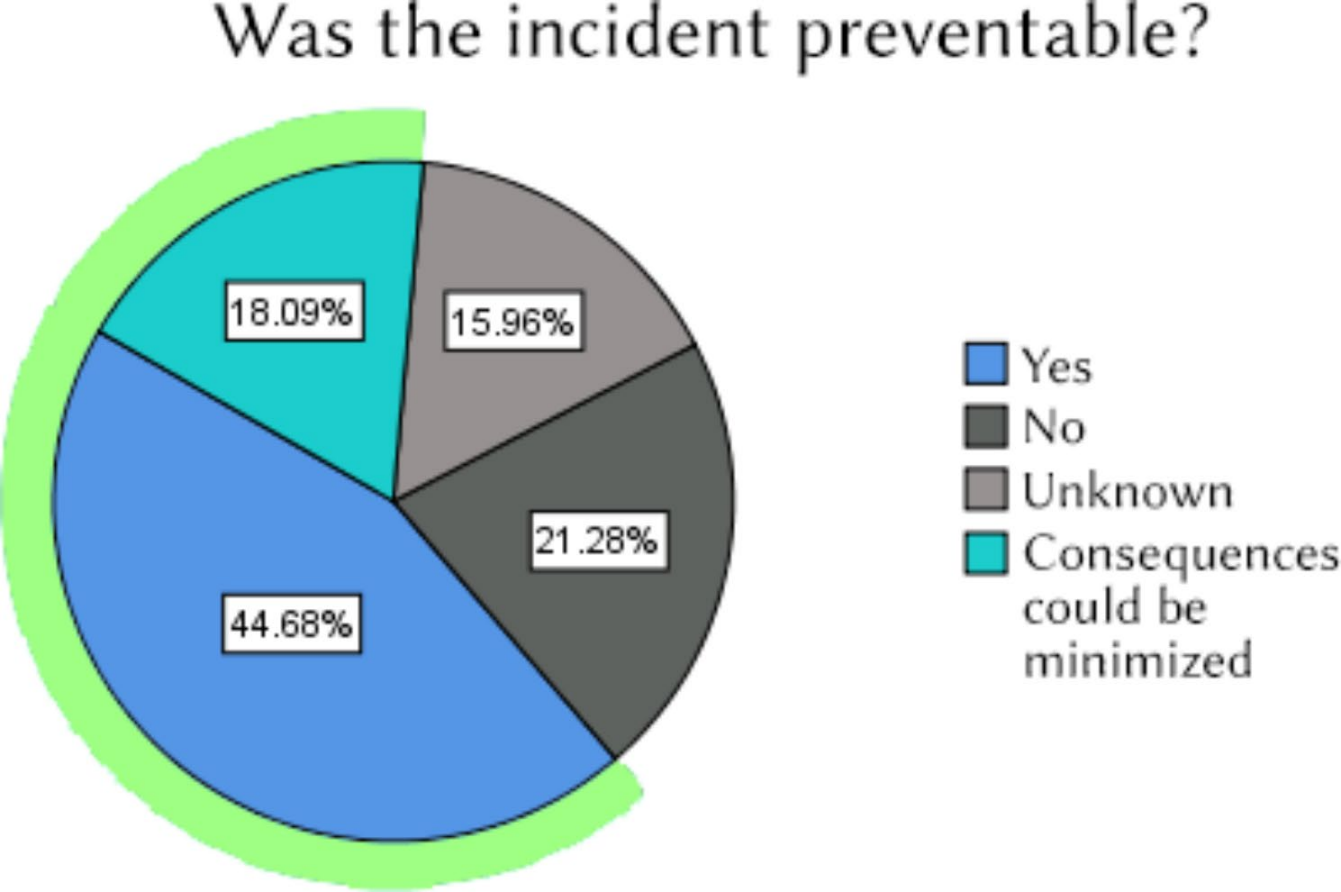
Preventability of the incident according to reporting physicians

18.09% of incidents were assessed by the respondents as entirely preventable. For 44.68% the consequences could be minimized, as per reporter’s assessment. (Fig. 2).

The most frequent failings contributing to the incident occurrence were: insufficient preoperative assessment (44%), incorrect interpretation of the patient’s state (33%), faulty manipulation technique (14%), miscommunication with surgical team (13%), and delay in emergency care (10%). Potential causes, as identified by reporting physicians, are presented in Fig. 1, highlighting the fraction related to anesthetic management, and the human factor.

**Fig. 3:**
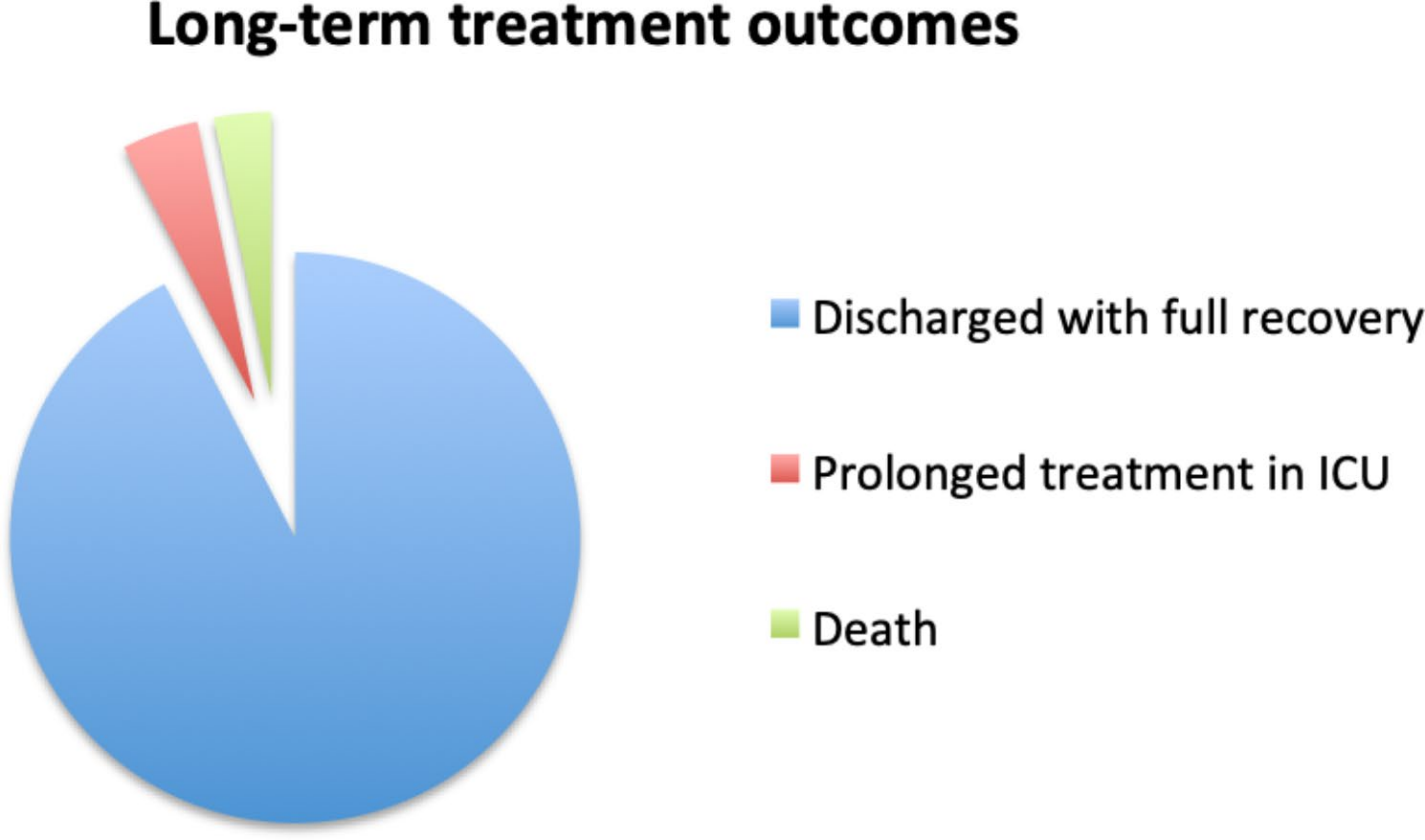
Long-term treatment outcomes

The consequences of the incidents were insignificant in over a half of the cases, but in 24.5% led to prolonged hospital stay, in 16% patients required an urgent transfer to the ICU, and 3% of patients died during hospital stay. The summary of long-term consequences for the patients is presented in Fig. 3. Prolonged ICU stay was defined as ICU stay exceeding the preoperatively planned period, due to a complication, caused by the incident.

The patients, who had an unplanned transfer to the ICU, prolonged ICU stay or died during hospital stay, were analyzed separately. We found that urgent surgery (OR 12 95 CI 5–32, p < 0.001), and surgery during night or at weekends (OR 9 95 CI 4–23, p < 0.01), as well as higher ASA status were predictors for these incidents to occur. Death was reported as a long-term treatment outcome, and, in majority of cases, was not directly caused by the incident, but mostly occur due to the patient’s severe condition.

**Fig. 4:**
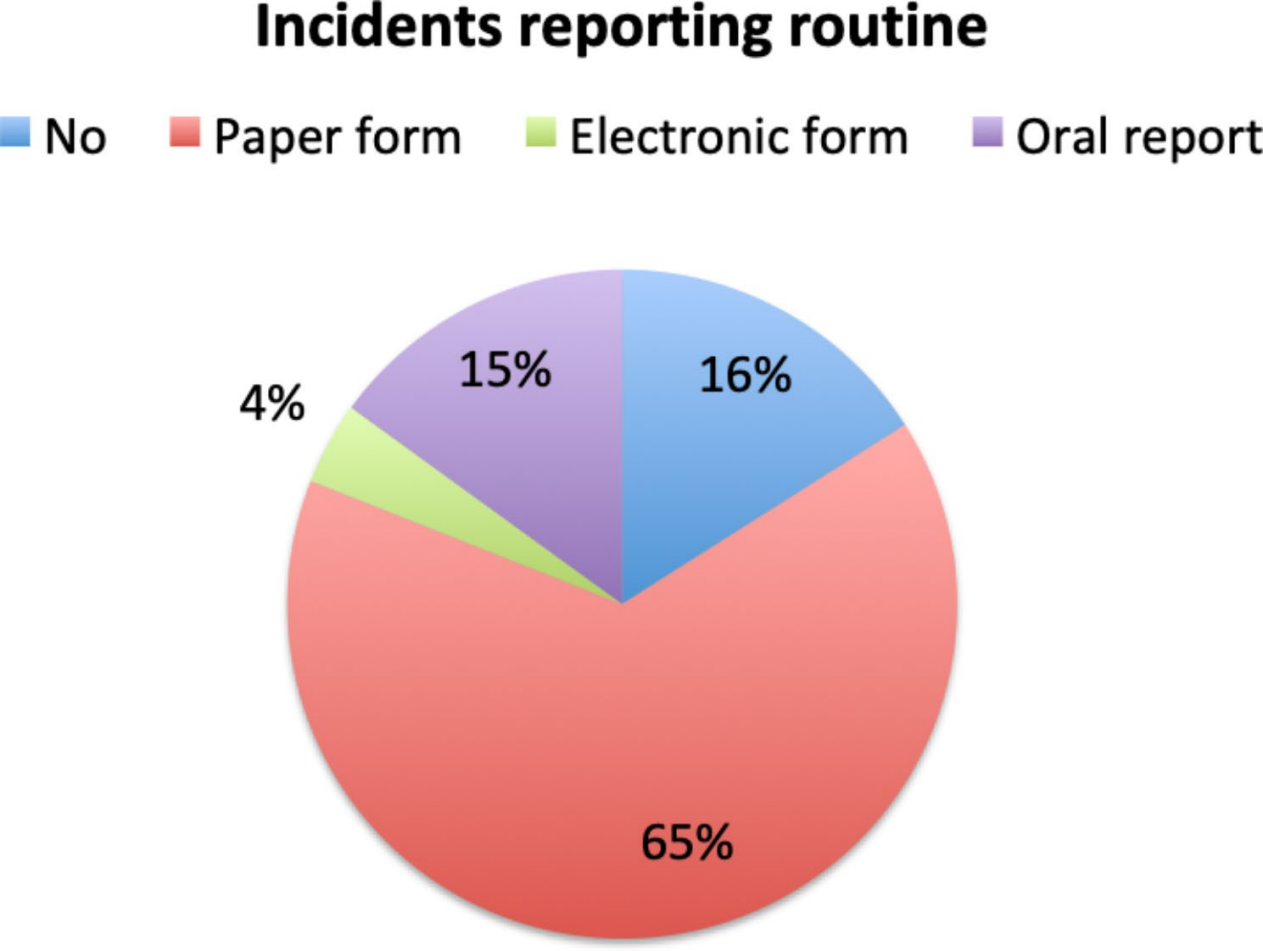
Incident reporting routine in responder’s hospitals

Regarding incidents reporting system, the majority of responders used paper forms (65%), some others – oral reports (15%), 4% used an electronic form, and 16% still followed no incident reporting routine (Fig. 4). The critical incident was followed by a detailed analysis within the department in 58.1% of cases, with 6.5% resulting in a permanent policy change.

## Discussion

We found that the incidence of critical incidents was 9.35 cases per 1000 anesthetic procedures, or 0.93%.

The higher age and ASA status were associated with critical incidents. However, a significant quantity of incidents involved patients assigned ASA II (45%), suggesting that the risks for this group may be currently underestimated. Another finding is that 73.4% of reported incidents occurred during daytime on weekdays, which are traditionally considered “safer”. These results could be explained by higher tendency to detect and report events in the daytime. Another reason is that the majority of surgeries and procedures are performed during daytime on weekdays. However, significant negative impact of the incident (unplanned transfer to the ICU or prolonged ICU stay) was associated with urgent surgery, surgery during night or weekends, as well as higher ASA status. These results could be explained by human factors like fatigue, lack of personnel, and highly qualified specialists during nighttime and weekends.

Reporting is one of the central components in patient safety improvement. Since the draft WHO guidelines for adverse event reporting and learning systems were published [12], different health care settings have been established regarding critical incident data collection and analysis systems. However, many countries still don’t have an organized national incident reporting and learning system, while others do not use them to their full potential, reporting as little as 7–15% of incidents [1, 13]. Another problem, prevalent in low- and middle-income countries, is the inability to provide full safety measures of patient monitoring. Therefore, knowledge of the incidence, circumstances, risk factors, and outcomes is important.

When comparing our results to other studies, reported incidence of critical incidents during anesthesia fits within commonly reported ranges. The frequency of incidents varies from 0.28 to 6.5% [9, 11]. A great variability could be explained by heterogeneity in the definitions, and the reporting method/criteria. Certain studies involve a dedicated supervisor [14], observing and documenting all activities, while others rely on self-reporting [4, 15]. The low rate of reporting could also be explained by underreporting, which is common. Studies have found that reporting systems detect 7–15% of incidents or adverse events [13]. This depends mainly on the culture, whether incidents are used as an opportunity to learn, or to blame.

Other authors also report that emergency surgery did not increase the risk of critical incident, while nighttime and higher ASA status did [9].

In our study, regional anesthesia decreased the risk of critical incidents. Other authors reported similar results [14, 15]. This could be explained by avoidance of regional anesthesia in patients with severe comorbidity or critical illness. Procedural sedation increased the risk of critical incident, compared to general anesthesia. The explanation we suggest is that the standard for minimal monitoring during procedural sedations is lower than for general anesthesia.

The emergencies were most commonly related to airways, unexpected massive hemorrhage and hemodynamic disturbances, and occurred mainly in the maintenance or induction phases of anesthesia. Other authors also report high incidence of respiratory and cardiovascular types of emergencies [11], which are commonly contributed to by the surgery or procedure itself. Critical incidents relating to airway management have been described in 17–34% of all incidents [18], cardiovascular – in up to 40% [10].

In our study most of the incidents had no negative impact on the patient outcomes, although 5.2% patients required prolonged ICU treatment and 3,2% died. The mortality was associated with anesthesia in 1 case per 20,100 anesthetic procedures (0.50 per 10,000). Compared to other studies, the reported data are similar: general mortality varies from 4.5[11] to 11.9% [14], and anesthesia-related mortality in developed countries ranges between 0.12 and 1.4/10,000 anesthetic procedures [17].

The majority of responders admit that critical incidents could be prevented, or that their consequences could be minimized. Common causes of the incidents were patient status or comorbidities, as well as surgical and anesthetic approaches, human factor, and drug side effects. Among the contributing factors were: insufficient preoperative assessment, faulty technique, and miscommunication. Human error has been continuously reported as one of the main causes of anesthesia-related critical incidents [3].

Reporting plays a significant role in understanding, analysis and system modifications to mitigate the number of critical incidents and human errors. Over two-thirds of responders have already used an internal reporting system in their hospital – a promising result for a low-middle income country. On the other hand, electronic reporting systems are still rare, which could be due to the limited financial and personnel resources. Other authors also emphasize that reporting systems are generally underused for many reasons, such as fear of blame or litigation, increased workload, forgetting to report an incident, and feeling that incident reporting is not useful [19]. In response to these problems, many healthcare organizations have adopted an electronic incident reporting system to replace paper-based incident reporting systems, and avoid the usual delays that result from manual data entry [20].

Since most changes in the healthcare system occur gradually, such audits help identify the current situation and tendencies in its development. Critical incident reporting is a significant part of patient safety and outcome improvement. Web-based national reporting systems could allow for further tracking and analysis of incidents.

### Study limitations

All cases were submitted voluntarily and anonymously, making it impossible to accurately estimate the real amount of incidents, possibly leading to underestimation. Different doctors define critical incidents in a different way [21], and also have a tendency to selectively report only major, interesting, or unusual events [22]. Moreover, some of the incidents could appear due to surgical manipulation (IVC syndrome), or as the anesthesia side effects (hypotension, bradycardia), and no clear objective criteria in the audit could be used to term them critical.

This audit did not account for the incidents, which have occurred during postoperative recovery.

Pediatric patients (age < 18 years) and patients over the age of 75 years were not included in the study.

The broad definition of “regional anesthesia” lacked subcategories (peripheral nerve blocks, spinal anesthesia etc.) in the form, making impossible to establish their individual contribution to the data. Certain adverse events, occurring during regional anesthesia (namely vessel puncture and bleeding) were also insufficiently distinguished in the form.

The degree of severity of reported incidents was not recorded, making it unclear, whether they could be truly classified as “critical”. This flaw is further augmented by the reliance of our design on individual judgment of respondents when classifying an event. Notable example of it is the “arrhythmia” event, which makes no distinction between benign and clinically significant rhythm disturbances.

Incident preventability was assessed by the reporting physician relying entirely on his subjective judgment, with no objective criteria provided in the study design.

Only minimal data on treatment of post-critical incident complications were collected, and no record of the specific complications, which occurred and required ICU stay, was made. For the lethal cases, the cause of death was not specified.

Reported data regarding the relative quantity of incidents occurring during different times of the day was collected as an additional parameter and cannot be used to draw any conclusions on this matter. Studies with methodology focused on this question report a higher rate of perioperative adverse events during nighttime surgery [23, 24].

## Conclusion

Critical incidents during anesthesia occur rather often, mainly during the induction or maintenance phase of anesthesia, and could lead to prolonged hospital stay, unplanned transfer to ICU and death. Reporting and further analysis of the incident are crucial, so we should continue to develop the web-based reporting systems on both local and national levels.

## Data Availability

The datasets used and/or analyzed during the current study are available from the corresponding author on reasonable request.

## Abbreviations

ASA: American Society of Anesthesiologists
ECG: Electrocardiography
LA: Local anesthetic
ICU: Intensive care unit
HR: Heart rate
SAP: Systolic arterial pressure
AP: Arterial pressure
